# Analyzing the Performance of the Population Health Environment on the Promotion of Provincial Governors in China

**DOI:** 10.3389/fpubh.2021.721492

**Published:** 2021-08-02

**Authors:** Hsiao-Fen Hsiao, Meng-Han Zhao, Wen-Ju Liao

**Affiliations:** Newhuadu Business School, Minjiang University, Fuzhou, China

**Keywords:** population health environmental index, economic growth, promotion of government officials, environmental degradation, Chinese economy

## Abstract

China's economy has achieved rapid growth, but the change has also brought about serious environmental degradation, which is the main factor endangering human health. This study empirically investigates the impact of the population health environmental index on the promotion of provincial governors using an ordered probit model. The sample of the study consists of regions where provincial governors, municipal mayors, and autonomous region chairmen were stationed from 1995 to 2015. The results show that the population health environmental index had a significant and positive impact on governors' promotions, especially in the eastern region. The reformation of the population health environmental index assessment system for government officials was the initial factor that brought about these effects.

## Introduction

China's economy has experienced rapid growth for almost four decades. Between 1979 and 2017, China's GDP grew at an average annual rate of 9.60%, and its per capita gross domestic product (GDP) grew at an average annual rate of 8.50%. The onset of the “Chinese Miracle” led to an expansion in China's total economic output, making it one of the fastest growing countries worldwide. The main factors and driving forces behind China's rapid economic growth are the government's push for maximum participation, encouragement, and control of the market and its emphasis on the promotion of a market economy. However, it is undeniable that because of economic growth, an increase in population health environmental pollution has become a serious threat. Statistics show that between 2004 and 2013, the cost of population health environmental degradation increased from 511.83 billion yuan to 2054.79 billion yuan, accounting for 3.3% of the total regional GDP. The cost of virtual management has also risen from 287.44 billion yuan to 697.33 billion yuan, an increase of 142.6%, because of the rising population health environmental costs of economic development. At the same time, the health problems caused by environmental pollution are gradually emerging. The UN Global Environmental Outlook report states that one-quarter of the global pre-mature deaths and major diseases are caused by man-made pollution and environmental damage. Population health environmental risk factors, especially air and water pollution, are also important reasons for the high incidence of tumors in China.

Some scholars have sought rational explanations behind the factors leading to the promotion of government officials. China has long used GDP growth as the main basis for these promotions. The political market in which these officials operate has a strong lock-in effect, which makes it difficult for them to find other job opportunities once they leave the political job market. While facing a dilemma between surrendering their rights and retaining them, government officials are forced to take actions that stimulate economic growth in their jurisdictions to gain promotion opportunities. China's current system of administrative decentralization gives local officials strong influence and control over local economic development. Local governments have free rein over important resources such as administrative approval, land acquisition, loan guarantees, and various preferential policies. To gain promotion opportunities, local governments will use the resources they have at their disposal for economic development and invest more in heavy industries. The local government will take the initiative to lower population health environmental protection thresholds and environmental regulations so that they can provide protection to sewage enterprises and attract foreign investments in the future. This has led to a series of population health environmental problems, such as wasting resources, air pollution, and soil degradation, and greatly endangers people's health.

In light of the increasingly serious problem of population health environmental pollution, the “Decision of the State Council on Implementing Scientific Viewpoint of Development and Strengthening Environmental Protection” in December 2005 explicitly included population health environmental protection in the assessment system of leadership teams and leading cadres. It also made the assessment a basis for the selection and appointment of leaders, as well as a basis for their rewards and punishments. In May 2007, the State Council circulated the “Notice on Issuing the Comprehensive Working Schemes on Energy Conservation and Emission Reduction.” It states that achieving energy conservation and emission reduction targets should be an aim in the comprehensive assessment system of local economic and social developments. These should also be included as key elements in the comprehensive assessment and evaluation of government-led cadres. Furthermore, the notice included the implementation of a “one-vote veto” system. The new “Environmental Protection Law of the People's Republic of China,” which came into effect in 2015, also emphasizes the need to use the achievement of population health environmental protection goals as an important basis for the assessment and evaluation of local government officials. The 18th National Congress of the Chinese Communist Party made the construction of an ecological civilization one of the focal points of China's modernization. It emphasized the inclusion of resource consumption, population health environmental damage and ecological benefits in the national economic development assessment system and the establishment of assessment methods and incentive mechanisms that reflect the requirements of an ecological civilization.

At present, population health environmental protection has been given an important position in relation to national development. To make the population health environmental index assessment of local officials work, it is necessary to link the population health environmental index with the selection and appointment of local officials. Local officials strive to get promoted, which also serves as a personal development goal for them. Therefore, linking the population health environmental index with promotions is the key to providing population health environmental incentives for government officials by realizing sustainable development goals.

This paper, on the basis of theoretical analysis, examines the regions of the provinces from 1995 to 2015 and studies whether such influences differ between different provinces. Our empirical results show that ecological and population health environmental indices have been integrated into the assessment and competition system for governors and that the improved population health environmental index has had a significant positive effect on the promotion of governors. Moreover, the positive effects of this improved population health environmental index on governor promotion are more pronounced in the eastern region than in the western region.

The contribution of this study lies in the fact that the existing literature only analyzes the impact of population health environmental protection on the promotion of officials in terms of the degree of population health environmental pollution investment in the population health environment. This study combines the two indicators and constructs a comprehensive index that uses data envelopment analysis to characterize the population health environmental index in each province, which deepens the understanding of the promotion assessment system of officials from an empirical aspect.

The structure of the study is as follows: the second part is the theoretical analysis; the third part is the construction of the model, data sources, and descriptive statistics of the variables; the fourth part is the analysis of empirical results; and the last part is the conclusion of the study.

## Literature Review

### Promotion of Officials and Economic Growth

Although China's economy has experienced rapid growth, certain growth conditions emphasized by economic growth theory, such as natural resource endowment, physical and human capital accumulation, and technological innovation capacity, are not unique to China compared to other countries. Some scholars (1–3) have explained the reasons for China's economic growth from the perspective of the government system. They noted that administrative decentralization and fiscal decentralization reforms, which consist of financial responsibility, motivate local governments to maintain market order and promote local economic growth (1–3).

Easterly (4) stated that growth required the “appropriate incentive” because people respond to it, and any factor that affects that incentive will have an impact on economic growth. Zhou (5) believed that conducting “competition for promotion”-based GDP growth for local officials was one of the most important means to explain government incentives and economic growth. China's M-shaped economic structure allows for a considerable degree of comparability of the economic performance across provinces and regions. Competition among local officials to maintain their positions becomes a norm with the presence of centralized personnel administration power at the central and higher levels of the government. Li and Zhou (6) studied data on Chinese provincial officials from 1979 to 1995 and concluded that there exists a correlation between economic growth and the probability of provincial officials being promoted during their tenure. They extended the data sample to the period of 1979–2002 and verified the central government's adoption of a related performance assessment system that was focused on the economic performance of provincial officials (6, 7). Similar points were made by Xu and Wang (8), Feng and Wu (9), and Wu and Chen (10), among others.

However, some scholars have called into question the logic behind the theory of “competition for promotion.” Tao Ran et al. (11) conducted an empirical re-evaluation of the panel data of Chinese provincial officials from 1979 to 2002. They found that there was no official assessment system that directly links economic growth with political selection. Moreover, they pointed out that under everchanging central-regional and government-enterprise relationships, the incentives of local governments to maximize fiscal revenue explain the economic growth in China's transformation process (11).

Opper and Brehm (12) were the first to explain government officials' promotions from the perspective of political network strength. They constructed a “network relationship index” based on the interrelation of the three aspects of hometown (born in the same province), school (graduated from the same school), and colleague (worked in the same administration), which represented the strength of the relationship between provincial officials and the Politburo Standing Committee. The researchers found a positive correlation between the index and the probability of government officials receiving a promotion when examining the data from 1987 to 2005 (12). The empirical analysis of Shin et al. (13) also shows that factional ties to senior leaders are the key to determining promotions.

Although the academic community has not reached a consensus on whether economic performance affects the promotion of government officials, the display of political achievements plays an important role in their political goals, mainly to protect their current positions and receive promotions in the future. These goals require a certain level of economic performance.

### Promotion of Officials and the Population Health Environment

During China's period of miraculous growth, local officials played an important role in promoting economic reform, strengthening regional cooperation, building infrastructure, developing the private economy, and attracting investment. Although the central government's assessment of local officials has shifted from a political performance-based approach to an economic approach to keep them motivated during their tenure, it has also brought about a series of negative effects, such as overlapping projects and cross-regional pollution.

The “Interim Provisions on Tenure of Leading Party and Government Cadres” emphasize that the provincial governor's term of office is 5 years. To make their performance noticeable in the short term, they tend to choose projects with highly visible results. In contrast, projects such as environmental protection, which are long-term and difficult to measure, are marginalized (14, 15). In addition, officials compete with each other to gain economic growth in their jurisdictions. For example, to attract foreign capital, local governments adopt tax incentives (16) and policies to protect businesses, even though these policies may come at the expense of the environment (17). Officials at all levels are more likely to invest in infrastructure to increase local GDP, but such investments tend to harm the environment and health; the greater the infrastructure investment is, the worse the environmental quality (18–20).

In recent years, the central government has recognized the importance and necessity of population health environmental protection and has explicitly combined the population health environmental index with the assessment system for officials. Some scholars have studied whether this incentive works. Kahn (21) found that since the Eleventh 5-year Plan (2006–2010) emphasized linking promotion opportunities for officials to water pollution, there has been a significant decrease in chemical oxygen demand emissions and an improvement in water pollution levels. Liang (22) studied data from 31 provinces in China from 2001 to 2010 and found that the inclusion of environmental pollution in officials' performance assessments resulted in a significant reduction in air pollutant emissions. In addition, Wu et al. (19) noted that mayors' and municipal party secretaries' probability of receiving a promotion was negatively correlated with their investments in improving the environment. Through empirical studies, Sun et al. (23) found that improvements in environmental quality and energy efficiency have had a positive effect on the promotion of mayors. These results indicate that the reformation of the performance assessment system for local officials has had a positive effect. Changing the impetus for promoting officials prompted them to strengthen the economic development of their jurisdictions, which is conducive to achieving green development. We consider the following hypothesis in this context:

Hypothesis 1: The population health environmental index within an official's jurisdiction during their tenure is positively correlated with their promotion.

China is a vast country, with major differences between the eastern and western regions of the country in terms of their geographical location, natural resources, and talent pool. Since the Chinese economic reform, China's economy has seen rapid development in the eastern regions and underdevelopment in the western regions. The more underdeveloped a region is, the more economic development is prioritized there, and thus the promotion of officials becomes more dependent on economic growth. It is difficult to consider the population health environmental index due to the limited resources available in these regions. In contrast, in the more economically developed regions, the promotion of officials is less dependent on economic performance but rather on more stringent regulation of the population health environment. This study examines the differences in the impact of the population health environmental index on the promotion of government officials in different regions based on the regional classification criteria issued by the National Bureau of Statistics. We consider the following hypothesis in this context:

Hypothesis 2: The difference in the regional population health environmental index has a significant positive correlation with the promotion of officials.

In November 2005, the central government issued the “Decision on Implementing the Scientific Outlook on Development and Strengthening Environmental Protection,” which made it clear that population health environmental protection is an important indicator in the promotion assessment of government officials. To investigate whether this policy works, this study analyzes the impact of the population health environmental index on the promotion of government officials. In this context, consider the following hypothesis:

Hypothesis 3: The inclusion of the population health environmental index in local assessment indicators will significantly affect the promotion of government officials.

## Research Models and Data Sources

### Model Specifications

Consider the following ordered probit model:

(1) $$\begin{array}{r} {\text{Promotion}}_{\text{it}}=\beta_{0}+\beta_{1}*{\text{PHEI}}_{\text{it}}+\beta_{2}*{\text{gdp}}_{\text{it}-1}+\beta_{3}*x_{\text{it}}+\varepsilon_{\text{it}} \end{array}$$

The model uses a panel data structure, with the annual province sample as the unit of analysis. The subscripts and represent province and year, respectively. The dependent variable is a ternary variable representing the promotion of provincial officials during their tenure, =2 represents a promotion, =1 represents no change, and =0 represents a demotion or other circumstances. The explanatory variable represents the population health environmental index of government officials during their tenure, gdp represents the average annual growth rate of GDP, is the set of control variables, and is the random error term.

### Research Sample and Data Sources

The variables used in this study were gathered from the population health environmental index, economic growth performance, and personal characteristics of government officials in 30 provinces, autonomous regions, and municipalities directly under the central government (excluding the Tibet Autonomous Region due to data unavailability) from 1995 to 2016. Governors, regional presidents, and mayors of municipalities (hereinafter collectively referred to as governors) were included in the sample.

**Figure 1:**
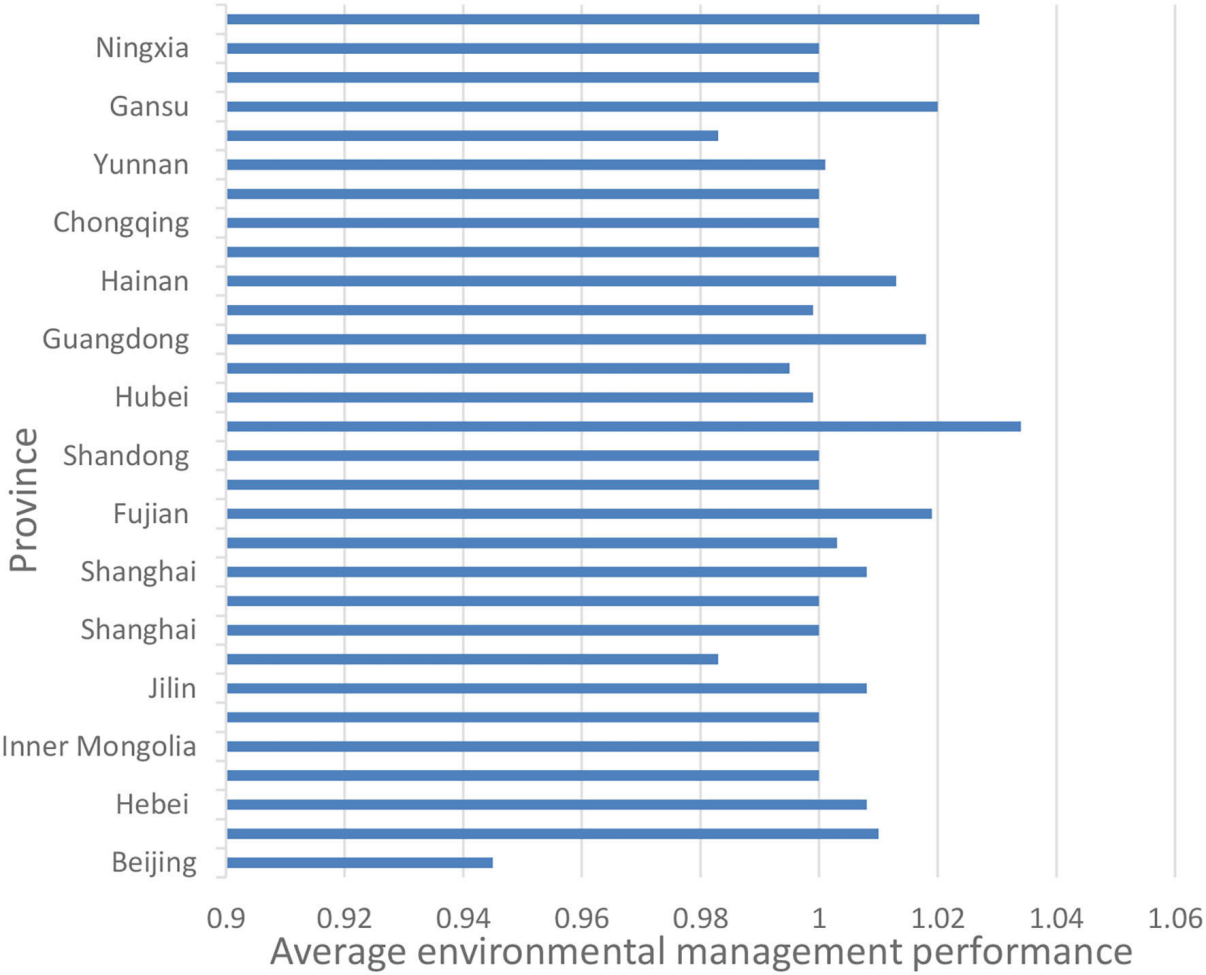
Average environmental health index of each province from 1996 to 2015.

Figure 1 represents the average environmental health index of each province in china during the period of 1996 to 2015. The population health environmental index data were obtained from the “China Statistical Yearbook,” provincial statistical yearbooks, “China Environmental Yearbook,” and “China Statistical Yearbook on Environment” issued by the Ministry of Ecology and Environment of China. Some of the missing data from the yearbooks were supplemented by the “Annual Report on Environmental Quality Conditions” from each province.

The economic data for the period of 1995 to 2004 were derived from the “1952-2004: Data of Gross Domestic Product of China,” adjusted by the National Bureau of Statistics according to the national economic census. The GDP data were taken from the “China Statistical Yearbook.”

Personal data such as the age, tenure, and educational background of local officials were gathered from their official biographies on Xinhuanet and People's Daily Online and search engines such as Baidu.

### Variables

#### Variables for Promoting Government Officials

Zhou (5), Wang and Xu (24), and Tao et al. (11) delineated the destinations of provincial governors as follows. (1) Transfer to the Central Government, that is, appointed as a national leader or to a regular position, such as Premier, Vice-Premier, member of the Politburo Standing Committee, Secretary of the Central Secretariat, Chairman/Vice Chairperson of the Chinese People's Political Consultative Conference, Chairman/Vice Chairperson of the National People's Congress, Head of the United Front Work Department, Minister responsible for the National Development and Reform Commission, and other positions in a central ministry or department. (2) Promotion to another province, that is, to become the secretary of that province. (3) Promotion in their current province, that is, to become the secretary of their current province. (4) A lateral transfer, that is, a promotion to another province, serving as the governor of their current province, or promotion to a deputy post in a central ministry. (5) Retirement to second-tier positions, that is, no longer holding substantive party or political positions, but only deputy positions in bodies such as the National People's Congress or the Chinese People's Political Consultative Conference, among other positions. (6) Demotion, that is, to serve as the chairman of the Chinese People's Political Consultative Conference of either their current or another province, or to serve as the chairman of the Standing Committee of the National People's Congress, among other positions. (7) Incumbent, that is, the official will still be in office at the end of 2015. (8) Others, which include samples of disciplinary violations, deaths, or unknown whereabouts of individuals. A transfer to the central government or a promotion to the post of secretary of the current or another province is considered a promotion and has a value of 2. A lateral promotion has a value of 1, and other cases have a value of 0.

#### Population Health Environmental Index

In this study, data envelopment analysis (DEA) was employed to measure the population health environmental level of officials from various provinces during their tenure. DEA is a non-parametric estimation method created by Charnes and Cooper in the 1970s. It is widely used in the field of performance measurement and has gained significant popularity in its application for measuring population health environmental indices in recent years (25, 26). The DEA model has many advantages. Assumptions regarding the production function are not required. It only has to measure the linear programming based on the corresponding input output data to obtain performance levels. At the same time, it can compare the efficiency changes of different decision-making units in different periods. The DEA-Malmquist index used in this paper is an improvement on the existing static DEA. It can compare the efficiency of different decision-making units in the same period, compare the efficiency changes of the same unit in different periods, and analyze the overall efficiency to determine the cause of efficiency changes.

For the purpose of conducting performance measurements, each province and district is considered a decision-making unit. The Malmquist index of the population health environmental index by region computed as an output-oriented calculation for each province and district as a decision unit from period t to t+1, assuming constant returns to scale, is as follows:

(2) $$\begin{array}{r} M_{0}\left(x^{t},\;x^{t+1},\;y^{t},\;y^{t+1}\right)={\left[\frac{D_{0}^{t}\left(x^{t+1},\;y^{t+1}\right)}{D_{0}^{t}\left(x^{t},\;y^{t}\right)}*\frac{D_{0}^{t+1}\left(x^{t+1},\;y^{t+1}\right)}{D_{0}^{t+1}\left(x^{t},\;y^{t}\right)}\right]}^{1/2} \end{array}$$

In Formula (2), t and t+1 represent two adjacent years. (*x*^*t*^, *y*^*t*^) and (*x*^*t* +1^, *y*^*t* +1^) denote the investment and production input output vectors in year *t* + 1. ${\;D}_{\;0}^{\;t}$ and${\;D}_{\;0}^{\;t\;+1}$ denote the distance function based on the technology in year *t* for both years. When >1, the overall efficiency of the population health environmental index in the region improves, while the opposite is true when <1. At the same time, the Malmquist index can be further broken down into technical efficiency (TE) and technological change (TC):

(3) $$\begin{array}{r} M_{0}\left(x^{t},\;x^{t+1},\;y^{t},\;y^{t+1}\right)=\frac{D_{0}^{t+1}\left(x^{t+1},\;y^{t+1}\right)}{D_{0}^{t}\left(x^{t},\;y^{t}\right)}\\ \;*{\left[\frac{D_{0}^{t}\left(x^{t+1},\;y^{t+1}\right)}{D_{0}^{t+1}\left(x^{t},\;y^{t}\right)}*\frac{D_{0}^{t}\left(x^{t+1},\;y^{t+1}\right)}{D_{0}^{t+1}\left(x^{t},\;y^{t}\right)}\right]}^{1/2}\\ \;=\text{TE}*\text{TC} \end{array}$$

In Formula (3), TE examines the change in the efficiency of the region from t to t+1 relative to the production possibility frontier, and TC reflects the change in the average technological level in all the regions during the period of change. Similarly, a TE or TC >1 indicates a technology efficiency improvement, and the opposite is true when it is <1.

In this study, the amount of investment in population health environmental pollution control and the total number of personnel in the population health environmental protection system at the end of the year are considered as the input after taking into account the number of funds and the human resources invested in the population health environmental index. Industrial wastewater emissions, industrial sulfur dioxide emissions, and industrial smoke or dust emissions are regarded as the outputs.

#### Economic Growth Performance

All data on GDP in this study have had the effects of inflation stripped away, and the average GDP growth rate during an official's tenure was used as the measure of a region's economic growth performance. The study also uses the lagged values of these variables as the explanatory variables, thus avoiding any possible endogeneity problems.

The formula for calculating the annual average GDP growth rate is as follows:

(4) $$\begin{array}{r} g_{T}=\frac{1}{T}\sum_{t=1}^{T}g_{t} \end{array}$$

where T represents tenure, t represents year and *t* (*t* = 1,2. *T*), represents the GDP growth rate for year t, and represents the average annual GDP growth rate during the official's tenure.

#### Personal Characteristics of Officials

The personal characteristics of local officials include age, tenure, and educational background.

The classification criteria of Wang et al. (27) have been taken into consideration when determining a government official's tenure. If the official takes office between January and June, the same year shall be deemed to be the starting year of the tenure; if the official takes office between July and December, then the following year shall be deemed the starting year of the tenure. This means that if an official resigns between January and June, then the year before the resignation is recorded as the last year of the term; if the official resigns between July and December, then the same year is recorded as the last year of the term.

Age (age) is the difference between the year an official's position changes and the year of birth. In 1980, Comrade Deng Xiaoping abolished the lifelong tenure of leading cadres and implemented mandatory retirement, proposing that cadres should be younger, more knowledgeable, and more professional. As such, cadres appointed to party and government positions tend to be younger than those before. Therefore, the probability of an official receiving a promotion decreases if the age exceeds a certain threshold. In light of this proposition, the study sets a dummy variable to check whether the age is over 55 years (age 55). The value is 1 if the age is ≥55 and 0 otherwise. For the details of variables, please refer to Table 1.

**Table 1:** Description of variables.

**Variable name**	**Variable code**	**Variable definition**
Change of position	Promotion	Changes in official positions, a value of 2 represents promotion; a value of 1 represents no change; a value of 0 represents demotion or other cases
Environmental health index	PHEI	Based on DEA, a comprehensive index that characterizes and calculates the population health environmental index in each province
Environment health index_ Policy indicators	PHEII_policy	The interaction term between population health environmental index and the policy year dummy variable
Average annual GDP growth rate	Gdp	The average annual GDP growth rate during an official's tenure
Term of office	Tenure	An official's time in office
Age	Age	Difference between the year an official's position changes and the year of birth
Line at 55 years old	Age 55	Dummy variable: takes the value of 1 if the age is ≥55; takes the value of 0 otherwise
Education background	Degree	Takes the value of 0 for a college degree and below; takes the value of 1 for an undergraduate degree; takes the value of 2 for a post-graduate degree and above
Respective region	West	Dummy variable: takes the value of 1 for western province; takes the value of 0 otherwise
Policy effects	Policy	Dummy variable: takes the value of 0 from 1995 to 2005; takes the value of 1 from 2006 to 2015

### Descriptive Statistics

Table 2 shows the descriptive statistics of the variables that were considered in the analysis. A maximum population health environmental index score of 4.36 and a minimum of 0.031 indicate significant regional differences in the population health environmental index. Economic growth performance also varies considerably across regions. The highest average annual GDP growth rate is 18.92%, while the lowest is only 4.4%. In terms of tenure and age of governors, the average term of office is 5.09 years, with a maximum term of 12 years and a minimum term of only 1 year concentrated between November 2002 and March 2003. Since the 16th National Congress of the Communist Party of China was held in November 2002, there has been a massive reshuffling of leadership at the provincial level. The average age of provincial governors is 59.80 years, ranging from 46 to 66 years, with the majority over 55 years of age. The average value of educational background is 1.39, which indicates that most leaders hold a University degree or a higher degree.

**Table 2:** Descriptive statistics of the variables.

**Variables**	**Obs**	**Mean**	**Std. Dev**	**Min**	**Max**
Promotion	626	1.230	0.909	0	2
PHEI	599	0.982	0.402	0.031	4.358
Gdp	601	11.228	2.070	4.400	18.915
Age	630	59.803	4.013	46	66
Age 55	630	0.892	0.311	0	1
Tenure	630	5.087	2.051	1	12
Degree	630	1.394	0.613	0	2
East	630	0.367	0.482	0	1
West	630	0.367	0.482	0	1
Policy	630	0.524	0.500	0	1

### Correlation Analysis

The results of the Pearson correlation coefficient test among the values in this study are shown in Table 3. The correlation coefficient between the population health environmental index and the promotion of governors is 0.067, which is significant at the 10% level. Preliminary verification confirms Hypothesis 1, that is, the population health environmental index has a positive impact on governor promotion; the better the population health environmental index is, the greater the probability that the governor will be promoted. The relationship between economic growth performance and governor promotion is found to be insignificant, whereas literature from other sources confirms the opposite, and thus further research and analysis are required in this case. The control variables, the governor's age dummy variable, and tenure, are shown to be negatively correlated with promotion.

**Table 3:** Pearson correlation coefficient matrix.

**Variables**	**Promotion**	**PHEI**	**Gdp**	**Age**	**Age 55**	**Tenure**	**Degree**	**Policy**
Promotion	1.000							
PHEI	0.067*	1.000						
Gdp	−0.001	0.029	1.000					
Age	−0.467***	−0.014	0.027	1.000				
Age 55	−0.081**	−0.021	−0.072*	0.676***	1.000			
Tenure	−0.327***	0.024	0.084**	0.411***	0.170***	1.000		
Degree	0.051	0.008	0.148***	−0.122***	−0.111***	−0.015	1.000	
Policy	0.091**	0.001	0.291***	−0.025	0.024	−0.050	0.503***	1.000

***, **, and * indicate statistical significance at 1, 5, and 10 percent, respectively.

## Analysis of Empirical Results

### Base Results

Table 4 shows the regression results of the ordered probit model given in equation (1), showing the probabilities of a governor's promotion. In column (1), only the average GDP growth rate during the governor's tenure and the governor's personal characteristics have been added. The average GDP growth rate has a significant impact on a governor's promotion at the 1% level of significance; that is, the probability of a governor's promotion increases by 2.8% for a unit increase in GDP. This indicates that economic performance still plays an important role in officials' promotion. Generally, age has a significant negative effect on the probability of a governor receiving a promotion; that is, the older the governor gets, the less likely his chance of being promoted. However, with each additional increase after 55 years of age, the probability of a promotion increases by 53%, and the probability of a demotion is reduced by 50%, indicating that the probability of being promoted at the age of 55 is still high. Tenure also has a significantly negative effect on the promotion of governors. With a unit increase in tenure, the probability of getting a promotion decreases by 2.8%, suggesting that longer tenure is not a good sign and may reflect a lack of abilities. The impact of educational background on promotion is positively correlated at the 5% level of significance.

**Table 4:** Regression results of the ordered Probit model.

	**Basic result**		**Eastern region**	**Western region**	**Policy effects**
	**(1)**	**(2)**	**(3)**	**(4)**	**(5)**
	**Promotion**	**Promotion**	**Promotion**	**Promotion**	**Promotion**
PHEI		0.271* (0.153)	0.909** (0.422)	0.133 (0.204)	0.399* (0.239)
Gdp	0.123*** (0.034)	0.121*** (0.035)	0.232*** (0.075)	0.119** (0.054)	0.120*** (0.035)
Age	−0.289*** (0.027)	−0.289*** (0.027)	−0.362*** (0.052)	−0.343*** (0.045)	−0.289*** (0.028)
Age 55	2.354*** (0.308)	2.380*** (0.315)	2.116*** (0.612)	3.265*** (0.489)	2.381*** (0.318)
Tenure	−0.123*** (0.041)	−0.133*** (0.042)	−0.083 (0.077)	−0.121* (0.064)	−0.134*** (0.042)
Degree	0.239** (0.115)	0.284** (0.119)	−0.277 (0.215)	−0.072 (0.227)	0.279* (0.144)
PHEI_policy					−0.222 (0.312)
Policy					0.228 (0.344)
Cutoff point 1	−14.812*** (1.412)	−14.486*** (1.435)	−17.939*** (2.595)	−17.339*** (2.450)	−14.374*** (1.445)
Cutoff point 2	−14.208*** (1.401)	−13.891*** (1.423)	−17.475*** (2.573)	−16.360*** (2.420)	−13.778*** (1.434)
Sigma2_u:_cons	1.008*** (0.348)	0.981*** (0.341)	1.576* (0.941)	0.535* (0.298)	0.987*** (0.345)
Obs.	598	583	213	209	583

***, **, and * indicate statistical significance at 1, 5, and 10 percent, respectively.

*Figures in parentheses represent standard errors*.

Column (2) adds indicators that characterize the population health environmental index of governors. The average annual GDP growth rate and individual characteristics continue to be highly significant. The governor's population health environmental index during his tenure is positively correlated with his promotion at the 10% level of significance. For every increase in the population health environmental index by 1 unit, the probability of his receiving a promotion increases by 6.1%. This shows that the policy of incorporating the population health environmental index in the promotion assessment of officials has worked. Hence, Hypothesis 1 is accepted.

### Comparison of Different Regions

Considering the vast differences in the level of economic development and the degree of population health environmental pollution among the different regions of China, this study examines the influence of regional heterogeneity on the population health environmental index and the promotion of officials. Columns (3) and (4) of Table 4 present the regression results for the eastern and western regions. The results show that in the eastern region, the population health environmental index coefficient is significantly positive at the 5% level of significance; the better the population health environmental index is, the more it will aid in promoting governors. However, in the western region, the population health environmental governance performance coefficient is not significant. To some extent, this suggests that the positive effect of the population health environmental index on the probability of the governor receiving a promotion is more significant in the eastern region than in the western region. This is related to the difference in economic development between the regions. The eastern region is able to invest a part of its economic output to protect the environment due to its relatively high level of economic development, whereas promoting economic development is still the primary goal for the western region.

### Investigating Policy Effects in Stages

We observe the policy effects of the “Decision on Implementing the Scientific Outlook on Development and Strengthening Environmental Protection” issued by the State Council in December 2005, which explicitly makes population health environmental protection work an important indicator in the assessment of officials for promotions. The results of the interaction of the dummy variables of the population health environmental index and policy year represented by the interaction dummy variable PHEI_policy is shown in column (5) of Table 4. The coefficient on the population health environmental index (PHEI) is 0.399, which is significant at the 10% level of significance, and the coefficient of the interaction term PHEI_policy, which denotes the interaction between the population health environmental index (PHEI) and policy effect (policy), is −0.222 and is insignificant. Before the policy was issued in 2005, the population health environmental index already had a significant impact on the promotion of governors. After the promulgation of the policy, the positive impact of the population health environmental index on the probability of promotion of governors was strengthened, but not significantly. This indicates that the policy of incorporating population health environmental protection into officials' performance appraisals has played an initial role, but further monitoring is still needed.

## Conclusion

This study examines whether ecological and population health environmental indices are truly integrated into the assessment and promotion system of provincial governors by using a panel data analysis for 30 Chinese provinces (autonomous regions and municipalities directly under the central government) between 1995 and 2015. The study also examines the impact of regional heterogeneity and the enactment of population health environmental assessment policies on the promotion of governors. Our empirical results show that ecological and population health environmental indices have been integrated into the assessment and competition system for governors and that the improved population health environmental index has a significant positive effect on the promotion of governors. Moreover, the positive effects of this improved population health environmental index on governor promotion are more pronounced in the eastern region than in the western region. Finally, while the “Decision on Implementing the Scientific Outlook on Development and Strengthening Environmental Protection” issued in 2005 increased the negative impact that population health environmental pollution has on provincial governors, it is still not significant.

It has been well-documented that while the “GDP-only” performance assessment model allows local officials to promote economic development in their jurisdictions, it also brings about a series of population health environmental problems, such as energy consumption, air pollution, soil degradation and a high incidence of cancer. To reduce the worsening population health environmental problems, China issued documents such as the “Decision on Implementing the Scientific Outlook on Development and Strengthening Environmental Protection” in 2001, which explicitly included population health environmental protection in the promotion assessment system for officials.

Although the jurisdiction's economic growth performance still plays an important role in the governor's promotion assessment, ecological and population health environmental factors have also begun to play a vital part in the assessment, indicating that the policy of reforming the official promotion assessment system has achieved its intended initial results. Therefore, to build a green China, it is necessary to further improve the assessment system for government officials to refine various environmental health protection assessment indicators and to establish a long-term system for green promotion.

## Data Availability Statement

The original contributions presented in the study are included in the article/supplementary material, further inquiries can be directed to the corresponding author.

## Author Contributions

H-FH and M-HZ developed the theoretical formalism, performed the analytic calculations, and performed the numerical simulations. H-FH and W-JL contributed to the final version of the manuscript. All authors contributed to the article and approved the submitted version.

## Conflict of Interest

The authors declare that the research was conducted in the absence of any commercial or financial relationships that could be construed as a potential conflict of interest.

## Publisher's Note

All claims expressed in this article are solely those of the authors and do not necessarily represent those of their affiliated organizations, or those of the publisher, the editors and the reviewers. Any product that may be evaluated in this article, or claim that may be made by its manufacturer, is not guaranteed or endorsed by the publisher.
